# The Cytoskeleton In Vivo

**DOI:** 10.1371/journal.pbio.0020100

**Published:** 2004-04-13

**Authors:** Beatriz García Fernández

## Abstract

A comprehensive understanding of the cytoskeleton can only be achieved by the combination of biochemical, cellular, and whole organism studies

As a student I always marvelled at the sight of single cells in culture moving over artificial surfaces and exhibiting membrane ruffles and protrusions. However, while I found cultured cells fascinating I always wondered how cells are able to move and regulate their shape in the context of a whole organism where so many space constraints exist and where all cellular processes have to be tightly regulated. Some answers to my questions began to emerge in a paper written by Baum and Perrimon (2001), in which the authors showed the expression and regulation of the actin cytoskeleton and of actin binding proteins in a real epithelium.

The cytoskeleton is a meshwork of protein polymers extending throughout the cytoplasm. It not only provides structural support for the cell but also plays a central role in a range of dynamic processes from signalling to endocytosis and intracellular trafficking. A particularly clear example of this is the use of actin cytoskeleton as a “wool” for knitting multiple dynamic structures such as lamellae, filopodia, and stress fibres. These structures determine cell shape and also produce the driving force accompanying many types of cellular movements including muscle contraction and cell division. We know many details about some of the proteins that modulate the dynamics of actin in these structures. However, most of them have been found biochemically and their function has been elucidated primarily using in vitro and cell culture assays of actin assembly. What about these proteins in the context of a developing organism? How do cells generate a spatially and temporally ordered network of actin filaments represented at the tissue level? To answer these questions, we need to move to experimentally accessible multicellular organisms, such as Drosophila, which offers virtually unlimited possibilities as a model system for the genetic and molecular analysis of biological processes.

Baum and Perrimon (2001) analyzed the function of a number of proteins involved in actin dynamics within the context of a developing epithelium—the follicle cells that surround the germ line cyst during Drosophila oogenesis. These cells have a simple polarised arrangement of actin filaments, which provides a useful system to study the spatial organisation of the actin cytoskeleton.

Taking advantage of the ability to generate clones of cells lacking specific proteins, the authors identified new functional roles for actin regulators such as CAP (a Drosophila homologue of adenylyl cyclase-associated proteins), Enabled (Ena) and Abelson (Abl). These proteins had been well characterized in cell culture and in vitro studies, but little was known about their function in a developing organism. Clones of cells lacking CAP (Figure 1), a protein known to inhibit actin polymerisation, maintained their epithelial polarity but had higher levels of actin and defects in the apical actin organisation. This result indicates that the inhibitory activity of CAP is restricted to one side of the cells, thus demonstrating that actin dynamics can be independently modified at opposite poles of an epithelium. Ena, a member of the Ena/VASP family proteins that catalyse filament formation, and Abl, a protein kinase that binds CAP in mammalian cells, were found to work with CAP in this process. The authors proposed that CAP, Ena, and Abl regulate the level and spatial organization of actin in the follicle cells.

**Figure 1:**
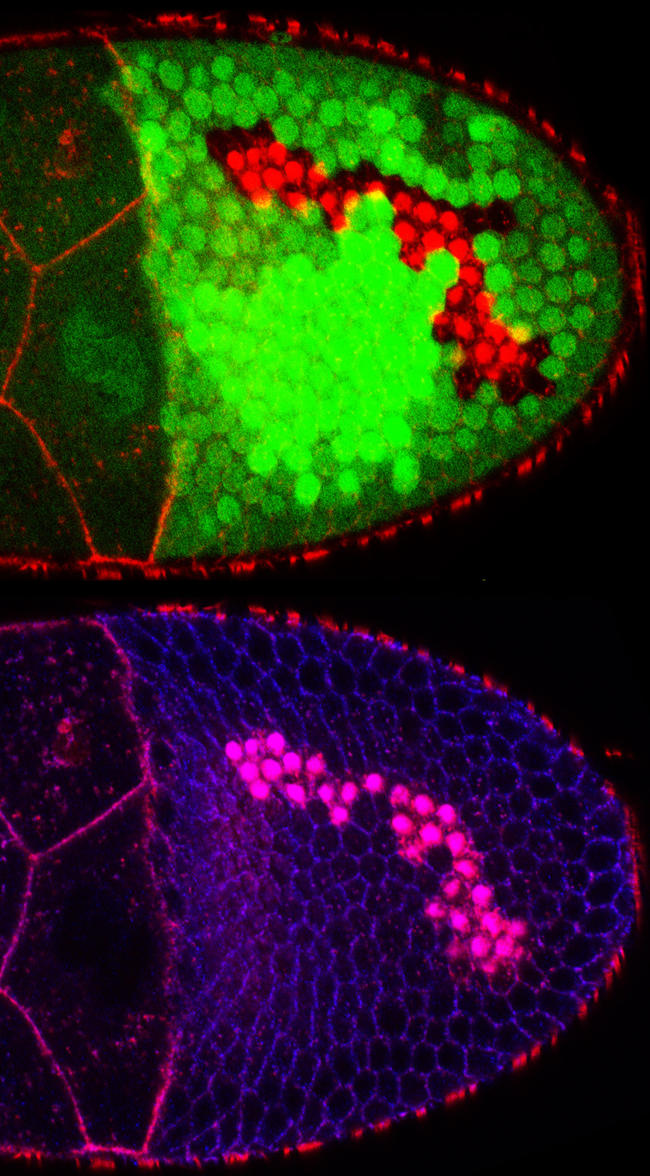
CAP Mutant Clones Follicle cells lacking CAP accumulate actin (red) in their apical region. Ena (blue in the bottom panel), also accumulates apically in the mutant cells (looks pink in the clone of cells due to overlap with F-actin in red). The mutant cell clones are identified by the absence of GFP (green in the top panel). Using this technique the cytoskeleton of mutant cells can be analysed in the context of a wild type epithelium. (Image kindly supplied by Buzz Baum.)

In contrast to the spatially restricted functions of CAP, Ena, and Abl, profilin and cofilin were shown to regulate actin filament formation throughout the cell cortex, a more global function that matches the results obtained in cell culture experiments. In summary, this study showed how proteins can organise actin in space and began to highlight some of the differences and similarities between cells in culture and in vivo. The functions revealed in the follicular epithelium were consistent with the roles previously shown in mammalian systems, but the experiments on intact tissue began to reveal a spatial and temporal functional dimension that could not have been observed in cell culture.

These experiments could be expanded to large-scale screens (St Johnston 2002), but this would be time consuming and could encounter the problem that some genes will be cell lethal, preventing the analysis of their function in actin dynamics. However, two more recent reports (Kiger et al. 2003; Rogers et al. 2003) describe a complementary and exhaustive search for regulators of cytoskeletal dynamics by taking advantage of genomic resources and the powerful RNA interference (RNAi) technique (Hutvágner and Zamore 2002). RNAi allows individual genes to be knocked out in a simple and controlled fashion.

Kiger et al. (2003) used RNAi in two different cell lines of Drosophila to screen a number of genes involved in signalling and cytoskeletal dynamics. They targeted 994 genes, of which 160 produced phenotypes in the experiment. The range of phenotypes varied from specific defects in the actin and tubulin cytoskeleton to others affecting cell cycle progression, cytokinesis, and cell shape. They also showed that only about 40% of the genes had similar loss-of-function phenotypes in both cell lines. This alone indicates an important limitation of many tissue culture experiments, since the same protein can have different effects depending on the cell type. Another valuable element of this work is that clustering of genes with similar phenotypes leads to the identification of pathways and networks of genes that are involved in cytoskeletal function.

Rogers et al. (2003), using only one Drosophila cell line, studied the effects of proteins involved in the formation of lamellae. The authors looked at the effects of loss of function in 90 genes known to be involved in actin dynamics and the formation and activity of the lamella. As well as confirming the function of many proteins already known to play a role in this process, this analysis allowed them to find interactions between genes and to build genetic pathways.

Together these two studies reveal that RNAi screens in tissue culture can be a powerful tool for finding new functions of known and uncharacterized genes, and new relationships between genes. However, this is only the beginning, and the genes identified in this manner will have to be tested in vivo, in systems like that of Baum and Perrimon, where specific functions can be assessed in time and space within the confines of real organisms. The focus must be to understand how all these molecular events and regulation cascades operate in individual cells to contribute to the generation of changes in a whole individual. Increasingly, the attention of developmental biologists is being drawn from genes and their products towards cells (Kaltschmidt and Martinez Arias 2002). The future, it seems to me, lies in the combination of in vitro systems, cell culture, and in vivo studies. I hope to apply this view in my analysis of the process of dorsal closure in Drosophila embryos, as an example of how signalling pathways coordinate and regulate the activity of the cytoskeleton in the generation of shape and morphogenetic movements (Jacinto et al. 2002).

## Abbreviations

Abl: Ableson
CAP: Drosophila homologue of adenylyl cyclase-associated proteins
ConA: concavalin A
Ena: Enabled
F-actin: filamentous actin
RNAi: RNA interference
